# A Single Dose of Dendrimer B_2_T Peptide Vaccine Partially Protects Pigs against Foot-and-Mouth Disease Virus Infection

**DOI:** 10.3390/vaccines8010019

**Published:** 2020-01-10

**Authors:** Rodrigo Cañas-Arranz, Mar Forner, Sira Defaus, Patricia de León, María J. Bustos, Elisa Torres, Francisco Sobrino, David Andreu, Esther Blanco

**Affiliations:** 1 Centro de Biología Molecular “Severo Ochoa” (CSIC-UAM), 28049 Madrid, Spain; rcannas@cbm.csic.es (R.C.-A.); pdeleon@cbm.csic.es (P.d.L.); mjbustos@cbm.csic.es (M.J.B.); elisa.torres@cbm.csic.es (E.T.); 2 Departament de Ciències Experimentals i de la Salut, Universitat Pompeu Fabra, 08003 Barcelona, Spain; mar.forner@upf.edu (M.F.); sira.defaus@upf.edu (S.D.); 3 Centro de Investigación en Sanidad Animal (CISA-INIA), Valdeolmos, 28130 Madrid, Spain

**Keywords:** FMDV, peptide vaccine, single dose, amount, pig

## Abstract

Foot-and-mouth disease virus (FMDV) causes a highly contagious disease of cloven-hoofed animals whose control relies on efficient vaccination. We have reported that dendrimer peptide B_2_T, with two copies of FMDV B-cell epitope VP1 (136–154) linked through maleimide units to T-cell epitope 3A (21–35)], elicits potent B- and T-cell specific responses and confers solid protection in pigs to type-O FMDV challenge after two doses of peptide. Herein we now show that B_2_T evokes specific protective immune responses after administration of a single dose of either 2 or 0.5 mg of peptide. High titers of ELISA and neutralizing antibodies against FMDV were detectable at day 15 post-immunization. Likewise, activated T cells and induced IFN-γ response to in vitro recall with FMDV peptides were also detected by the same day. Further, in 70% of B_2_T-vaccinated pigs, full protection—no clinical signs of disease—was observed upon virus challenge at day 25 post-immunization. These results strengthen the potential of B_2_T as a safe, cost-effective candidate vaccine conferring adequate protection against FMDV with a single dose. The finding is particularly relevant to emergency scenarios permitting only a single shot immunization.

## 1. Introduction

Vaccination remains the most effective approach to prevent human and animal diseases [1]. In animal health, development of safe, cost-effective and marker vaccines, capable of telling infected from vaccinated animals (DIVA), remains a challenge for many diseases, particularly viral ones [2,3]. Conventional vaccines based on inactivated or attenuated viruses entail risks such as accidental escape or incomplete inactivation of infectious viruses, as well as possible reversion of attenuated into virulent forms. Subunit or epitopic vaccines represent an alternative that solves most such problems by excluding the infectious agent and allowing targeting to well characterized viral epitopes relevant for protection [4,5,6].

Foot-and-mouth disease virus (FMDV) is the prototype member of the *Aphthovirus* genus within the *Picornaviridae* family [7] and the etiological agent of FMD, a highly transmissible infection of pigs and other cloven-hoofed animals, with huge economic impact worldwide [8,9]. FMD underscores paradigmatically the challenge of finding alternative strategies to the classic vaccines still used to prevent this highly contagious disease [10]. The massive amplification and shedding of FMDV in infected pigs turns this species to a key epidemiological factor for the spread of the virus over the course of outbreaks in many regions of the world [11]. In addition, the growing numbers of domestic pigs worldwide, particularly in Asian countries, make the development of pig-suited FMD vaccines a strategic task.

Conventional FMDV vaccines based on chemically inactivated virus have allowed FMD control and eradication in some countries, although their manufacturing process—not upgraded over recent decades—poses significant biosafety concerns that have been related to occasional escape episodes of diverse consequence [12,13,14]. This risk plus other limitations, such as the need for a strict cold chain to preserve stability, and the use of updated vaccine strains, because of the high potential antigenic diversity of the virus, underlie the adoption of non-vaccination policies in FMDV-free countries, a controversial and by no means risk-free practice, as borne out by not infrequent outbreaks in those locations. In crisis scenarios of this kind [15], vaccines incorporating outbreak-relevant epitopes, eliciting protective responses and generated as a quick response to the epidemic, can become an invaluable emergency resource for FMD containment [16]. Among such emergency vaccines, those based on synthetic peptides [6] are particularly appealing because of their (i) total lack of biological hazard; (ii) possibility of displaying various epitopes on a single platform; (iii) DIVA compliance; (iv) efficient synthetic production and characterization as pharmaceuticals, and (v) no cold-chain required; easy transport and storage [17].

The main B-cell antigenic site in FMDV, located at the GH loop of capsid protein VP1 (residues ca. 140–160), is structurally continuous [18,19]. Linear peptides reproducing this loop, either alone or in combination with T-cell FMDV epitopes, have been shown to confer limited protection in natural hosts [20,21,22,23].

A substantial enhancement in immunogenicity can be achieved by multiple display of B- and/or T-cell epitopes on a single molecular scaffold [17] inspired on the multiple antigenic peptide (MAP) platform of Tam [24]. In an initial realization in this regard, a peptide spanning residues 21–35 of FMDV protein 3A [thereafter T3A], which delimit an immunodominant T-cell epitope in domestic pigs [25], was N-terminally elongated into a Lys tree to which four copies of a B-cell epitope (residues 140–158 of VP1; containing the RGD motif that mediates binding to integrins, the cell receptors) were covalently linked in a dendrimeric (branched) fashion. The sequence of the B-cell epitope corresponded to that of the epidemiologically relevant O/UKG/11/01 isolate, belonging to serotype O the most prevalent worldwide [26]. This multivalent construct (named B_4_T) elicited high titers of FMDV-neutralizing antibodies, activated specific T cells, and fully protected pigs against FMDV challenge [27]. Interestingly, a simpler version (i.e., two B-cell epitope branches) of the peptide vaccine candidate, termed B_2_T, also elicited potent specific responses and conferred solid protection in pigs to challenge [28] (Table 1). In both B_4_T and B_2_T trials, animals were immunized with two 2-mg doses of peptide (3 weeks apart from each other) before challenge.

As mentioned above, emergency FMD vaccines, eliciting protective responses upon a single shot, are particularly valuable in containing uncontrolled FMDV outbreaks [14]. The modular nature of B_2_T affords considerable versatility in the sequences that can be integrated into the constructions, either as part of a single molecule or as mixtures of different molecules. Thus, incorporation of VP1 GH-loop sequences from different FMDV isolates can modulate/enhance the protective spectrum of the candidates. This is particularly relevant because of the high FMDV antigenic diversity reflected in seven serotypes and many variants within each of them, which makes matching of vaccine strains with circulating virus a critical issue for vaccine efficacy [11,15]. On the other hand, the T3A sequence mentioned above is highly conserved among FMDV serotypes and therefore can evoke heterologous responses, again contributing to broaden the protective response conferred by B_2_T. In addition to versatile, readily adaptable responses to new virus threats, emergency vaccines require fast and cost-effective manufacturing programs, which are ideally met by the expediency and flexibility inherent to chemical synthesis production.

Herein we have explored the possibility of: (i) Eliciting protective responses in pigs upon administration of a single B_2_T dose, and (ii) reducing the amount of antigen required to elicit protective responses. Thus, we report results with both the 2 mg dose of previous experiments [28] and with a reduced 0.5 mg inoculum. Remarkably, the latter dose elicits a rapid immune response involving high titers of FMDV neutralizing antibodies and specific IFN-γ secreting T cells at 15 days post-immunization (dpi). In addition, solid protection is observed in 80% of pigs vaccinated with 0.5 mg B_2_T, with no clinical signs upon viral challenge at day 25 dpi. Taken together, our results highlight the value of B_2_T as candidate FMD vaccine for pigs in emergency scenarios.

## 2. Results

### 2.1. A Single B_2_T Dose Elicits Rapid Humoral Specific Responses Including FMDV Neutralizing Antibodies

**Figure 1:**
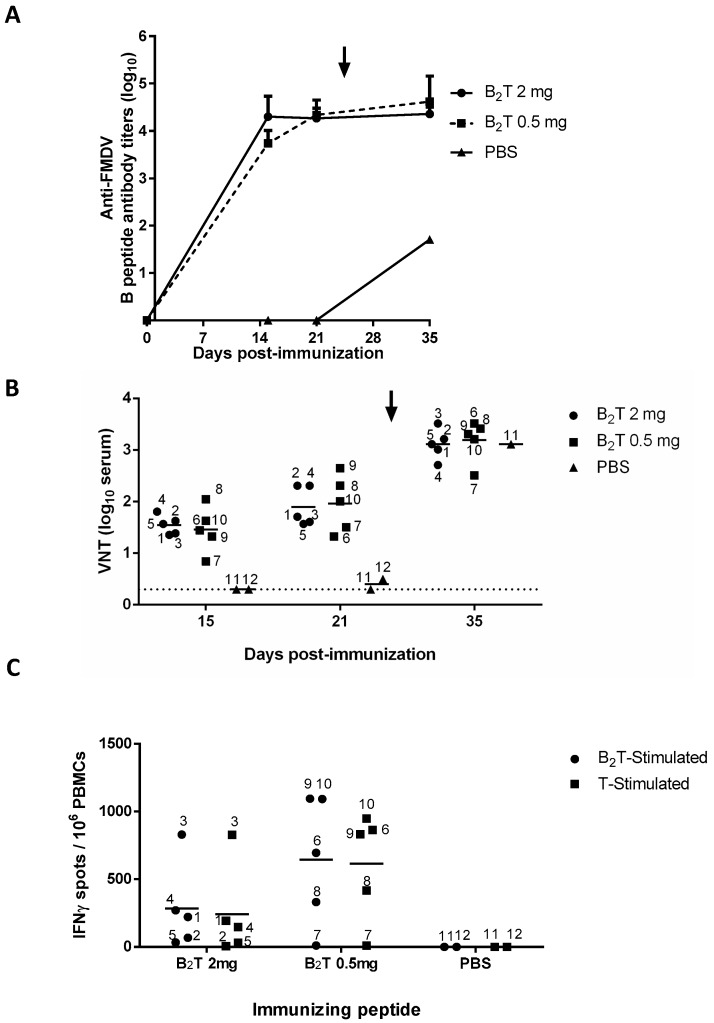
B- and T-cell responses in pigs immunized with a single dose and different amounts of B_2_T. Time course of the specific antibody responses in sera collected on the indicated days pi. (**A**) Total anti peptide B IgG titers analyzed by ELISA. Each point depicts mean antibody titers (calculated as described in Methods) ± SD for each group of pigs (*n* = 5). (**B**) Virus neutralization titers, VNT, expressed as the reciprocal log10 of the last serum dilution that neutralized 100 TCID50 of FMDV isolate O/UKG 11/01. Each symbol represents the value for an individual pig (numbering is included). Horizontal lines indicate the geometric mean for each animal group. In no case individual spontaneous reactivity was observed in the titers determined at day 0. Dotted lines denotes the assay detection limit. In (**A**,**B**) arrows point FMDV challenge (day 25 pi). (**C**) Specific T-cell responses measured by an ex vivo IFN-γ ELISPOT at days 15 pi. The frequency of FMDV-specific IFN-γ secreting cells was determined as detailed in Methods. Horizontal bars represent the mean frequencies of IFN-γ release spots of triplicates of peripheral blood mononuclear cells (PBMCs) from pigs stimulated in vitro with B_2_T (circles) or T (T3A) (squares) peptides (pig numbering is included).

Domestic pigs, in two different groups of five animals each, were immunized once with 2 or 0.5 mg of B_2_T (pigs 1 to 5 and 6 to 10, respectively), and two additional non-immunized animals were kept as controls (pigs 11 and 12 inoculated with PBS). Total FMDV-specific IgG antibodies were determined by ELISA at 0, 15, and 21 dpi. Both 2 and 0.5 mg doses elicited consistent and comparable IgG titers (log_10_) at 15 (4.3 ± 0.4 vs. 3.7 ± 0.3) and 21 (4.2 ± 0.2 vs. 4.3 ± 0.3) dpi (Figure 1A). Upon FMDV challenge (day 25) these titers were not boosted up, remaining similar in both B_2_T-immunized groups (4.3 ± 0.1 vs. 4.6 ± 0.5). Non-immunized control pig 11 that survived 10 days post-challenge (dpc) showed anti-FMDV titers >2log_10_ units lower than those of the immunized and challenged groups (Figure 1A).

Regarding induction of FMDV neutralizing antibodies, as observed with the ELISA results, no major differences were noticed between virus neutralization titers (VNT) in the groups immunized with either 2 or 0.5 mg B_2_T at day 15 (1.5 ± 0.2 vs. 1.4 ± 0.4) or day 21 pi (1.9 ± 0.4 vs. 2 ± 0.5) (Figure 1B). On the other hand, and in contrast to ELISA antibody titers, post-challenge VNT increased at 10 dpc (35 dpi) in both groups (3.1 ± 0.3 vs. 3.2 ± 0.4). As expected, no neutralizing antibodies were detected in the two control animals before challenge. The non-vaccinated animal that survived the challenge (pig 11), showed VNT (3.1) similar to those in immunized groups (Figure 1B), showing that neutralizing antibody levels in B_2_T-immunized/challenged pigs—including those found to be protected—were as high as those in infected and recovered animals.

### 2.2. B_2_T Elicits Early FMDV-Specific IFN-γ Responses

Specific T cell responses elicited by B_2_T at 15 dpi were determined by ELISPOT analysis of IFNγ-expressing PBMCs. High frequencies of spot-forming cells were found at day 15 in pigs immunized with either dose of B_2_T in response to in vitro recall with homologous peptide (284.8 ± 321.1 vs. 645.6 ± 467.9). On average, pigs in the 0.5 mg group showed higher frequencies of IFNγ-expressing PBMCs than those in the 2 mg group (Figure 1C). All responses were specific, as no peptide-driven IFN-γ-producing cells were detected in the two non-immunized pigs. PBMC stimulation with T3A peptide paralleled those observed with B_2_T (241.3 ± 365.8 vs. 614.4 ± 403.1) (Figure 1C), supporting the recognition of T3A as a T-cell epitope.

### 2.3. A Single Dose of B_2_T Peptide Confers Clinical Protection against FMDV Challenge

At 25 dpi, pigs in all three groups were challenged with FMDV. Animals were examined daily for clinical signs (see Methods) and considered protected when lesions were not observed or appeared only at the inoculation site [29]. As expected, PBS-inoculated control pigs 11 and 12 showed full FMD signs upon challenge, developing vesicular lesions on all four feet at 4 dpc and on the snout at 7 and 5 dpc, respectively (Table 2). The acute FMDV infection caused myocarditis, leading to heart failure and sudden death, of pig 12 at 5 dpc. In contrast, only 3 out of 10 peptide-immunized animals developed lesions outside the inoculation site: two pigs (3 and 5) in the 2 mg group and one (pig 10) in the 0.5 mg group. The remaining immunized animals did not develop any clinical signs, the cumulative lesion score of immunized groups being significantly lower than that of the non-immunized one (Table 2). In general, a correlation between lower body temperature and protection was observed, being the protected animals immunized with 0.5 mg B_2_T those that did not develop fever.

Detection of FMDV RNA in serum samples from challenged pigs by RT-qPCR showed the presence of viral RNA in non-immunized control pigs 11 (days 3 and 5 pc) and 12 (day 3 pc), as well as in the two immunized but non-protected pigs 10 (days 3 and 5 pc) and 3 (only at day 5 pc), which showed the higher lesion score at day 7 pc (Table 2). Hence, virus detection in sera associated with the severity of the lesions of challenged pigs. Further work is necessary to assess the potential contribution of viruses circulating in non-protected animals, and to a lesser extent in protected animals, to the spread of the disease under field conditions.

In conclusion, immunization with a single 2 mg dose and, remarkably, even more with a 0.5 mg dose of B_2_T dendrimer afforded substantial protection against conventional FMDV challenge.

## 3. Discussion

Protection after a single inoculation is a must for effective FMD vaccines, as it reduces both the cost of the vaccine as well as the logistics and labor expenses associated with the double immunization schedules. The application of a single dose vaccination program to the pig population can favor disease eradication, considering that pigs serve as amplifiers of FMDV and therefore a rapid control of the infection in swine is essential for disease control [11]. This is particularly relevant in settings such as diseases outbreaks occurring in areas where FMD is not enzootic and livestock remains unvaccinated [30]. To our knowledge, with the exception of adenovirus-vectored vaccines expressing FMDV empty capsids [31], few studies exist on the capability of FMD subunit vaccines alternative to classical ones to confer protection in relevant hosts after a single administration [32]. In this context, our optimization of the minimal amount of B_2_T dendrimer still conferring protection is pertinent, both for understanding the response mechanisms to B_2_T and for scaling down production costs, a relevant issue for FMD vaccine development.

We had previously shown that two 2 mg doses of B_2_T conferred solid protection to FMDV challenge in swine, and that protection correlated with the induction of strong B- and T-cell specific responses [28]. Here, we explore the feasibility of B_2_T as a protective, single dose subunit vaccine alternative to conventional FMD formulations. To this end, the original 2 mg dose and a four-fold lower one (0.5 mg) have been compared.

The challenge protocol followed in our experiment was as recommended by the OIE manual, for evaluation of protection against podal generalization (PPG) test, except that virus was inoculated 3 days earlier than indicated [33]. Under such conditions, full protection, considered as the absence of lesions at points other than the inoculation site, was observed in 70% of pigs vaccinated with B_2_T; 3 out of 5 in the 2 mg dose group, and 4 out of 5 in the 0.5 mg group. This level of protection correlated with an average increase in virus neutralizing (VN) antibodies that was not observed for B-specific IgG antibodies detected by ELISA (Figure 1), suggesting the recall upon viral infection of a subset of FMDV-specific memory B cells, as well as of memory T cells capable to promote B-cell maturation. This increment of VN antibodies in protected animals, matching that of a non-immunized pig, might reflect virus multiplication to a limited extent not resulting in detectable viremia (Table 2). Similar levels of VN antibodies boost upon virus challenge have been reported in pigs and cattle immunized with live-vectored vaccines or FMDV-like particles [34,35].

Non-protected (pigs 3 and 10) and partially protected (pig 5) animals showed lower levels of VN antibodies at the day of challenge, albeit animals with lower VNT yet protected were also observed (Figure 1B); the lack of correlation in a fraction of protected animals immunized with different FMDV vaccines has been described [21,36]. Experiments aimed at assessing the amount of antibodies that may target the FMDV receptor [37] as well as at examining cross-neutralization among other type O FMDVs will be foreseen to further characterize the antibody response evoked by the dendrimer peptide vaccines.

In addition, and supporting T3A recognition as a T-cell epitope, specific IFN-γ releasing activated T cells were detected rather early, at 15 dpi, in 7 of the 10 immunized pigs. The average frequencies of IFN-γ releasing cells in PBMCs from pigs in the 0.5 mg dose group were higher than those in the 2 mg group (Figure 1C and Table 2), possibly reflecting differences in the in vitro dose-effect of peptide stimulation and/or on the MHC allele composition of the animals analyzed. High doses of antigen might also facilitate its capture by APC/DC subsets with suboptimal costimulatory capacity that favor the development of regulatory T cells, which could limit the activation of B cells and subsequent antibody production as well as the development of IFN-γ producing cells [38]. In any case, additional studies of the effector mechanisms triggered by the dendrimers will be necessary to understand the effect on the protective responses of different peptide vaccine doses. One animal that was low responder at 15 dpi (pig 7), turned out to be protected upon challenge and pig 5, also low responder, was partially protected. Among responder animals, no clear correlation between IFN-γ releasing cell frequency at 15 dpi and protection to FMDV challenge was observed, i.e., non-protected pigs 3 and 10 showed high responses. Thus, as previously observed [21], VNT and T-cell stimulation do not fully correlate with the protection conferred by FMD peptide vaccines.

A potential problem of epitopic vaccines such us the dendrimeric peptides used in this study is the selection in non-protected animals of escape viruses capable to avoid antibody neutralization [21] and/or T cell recognition. In our experiment, we could not rule out that possibility since the limited number and size of the vesicles developed by non-protected animals did not allow virus recovery for sequencing analyses.

Our evidence of the level of full protection conferred by a single B_2_T dose upon challenge at 25 dpi is particularly remarkable with regard to the 0.5 mg dose group (80% of the pigs), as it opens the way to significant savings in manufacturing costs. Incidentally, on a related note, we have found that long-lasting (up to 19/20 weeks post-boost) reduced susceptibility to FMDV infection can be attained with two B_2_T doses and, remarkably, a similarly lasting protective response with a single dose of B_2_T (Cañas-Arranz et al., submitted).

Attempts to use B_2_T and B_4_T dendrimer peptides as vaccines in cattle showed a trend toward a reduced capacity to confer protection relative to swine, including the need for a third immunization to elicit protective levels on neutralizing antibodies [39,40]. Failure in conferring protection in cattle has been described for linear FMDV peptide vaccines containing a heterologous T cell epitope [41]. Further work on the requirements of the interactions between neutralizing epitopes and B cells, as well as the identification of new, effective T helper epitopes frequently recognized by individuals will contribute to the improvement of the effectiveness of this kind of vaccines for cattle and, eventually, other host species.

In summary, our findings portray the B_2_T peptide as a safe, potentially cost-effective candidate to be included in FMD vaccine formulations conferring single-shot protection in pigs. Formulation of dendrimers with alternative adjuvants as well as the incorporation of peptides corresponding to other T cell epitopes previously identified in pigs [42,43] can contribute to improve their immunogenicity in swine.

## 4. Methods

### 4.1. Peptides

Peptides identified as B- and T-cell epitopes of FMDV O/UKG/11/01 (Table 1) were assembled by Fmoc-solid phase synthesis, purified by reverse-phase liquid chromatography, and characterized by mass spectrometry. Dendrimeric B_2_T construct was conjugated in solution using two previously synthesized precursors: (i) B epitope (VP1, residues 136–154) plus a C-terminal Cys residue (free thiol form) and (ii) T epitope (3A, residues 21–35) elongated at the N-terminal with two Lys residues followed by an additional Lys branching derivatized as two maleimide groups. The B_2_T peptide was efficiently obtained after the thiol-maleimide ligation at pH = 6, RP-HPLC purification and MS characterization [27,28,44].

### 4.2. Virus

A virus stock derived from FMDV isolate O/UKG/11/01 [45] by two amplifications in swine kidney cells (IB-RS-2 cells) was used. The resulting virus maintained the consensus sequences at the capsid region [46].

### 4.3. Animals and Experimental Design

The immune response to B_2_T dendrimer peptide was assessed in ten 9-weeks-old white cross-bred Landrace female pigs (Agropardal SA breed), free of antibodies to FMDV. The study was approved (CBS2014/015 and CEEA2014/018) by the INIA Committees on Ethics of Animal Experiments and Biosafety, and by the National Committee on Ethics and Animal Welfare (PROEX 218/14). Pigs were randomly assigned to two groups of five animals each and immunized once (days 0) by intramuscular injection with 2 mL of Montanide ISA 50V2 emulsion (Seppic, Paris, France) containing 2 or 0.5 mg of B_2_T peptide. Two additional non-vaccinated pigs were kept as infection controls (#11 and 12). Animals were housed in separate units of the high-containment facility and challenged at day 25 with 1.6 × 10^4^ plaque forming units (pfu) of FMDV O/UKG/11/01, by inoculation at two sites of both main claws of the left-hindfoot pad (0.1 mL/site). Animals were monitored for clinical signs of disease during the 10 days (Table 2), and then euthanized at day 35.

### 4.4. Viral RNA Detection after Challenge

Serum samples were examined for the presence of viral RNA by real time RT-qPCR. Briefly, the cDNA obtained in a RT reaction using primer A (5′-CACACGGCGTTCACCCA(A/T)CGC-3′) [47] was amplified by qPCR using the “Light Cycler RNA Master SYBR Green I” kit (Roche, Basel, Switzerland) and LightCycler equipment following the instructions of the manufacturer. The amplicon synthesized spanned a conserved region of 290 pb length in the 3D protein coding sequence, and was amplified using primers A (5′-CACACGGCGTTCACCCA(A/T)CGC-3′) and B (5′-GACAAAGGTTTTGTTCTTGGTC-3′). The values for the quantification of the samples were obtained from a standard curve from a RNA transcript derived from the infectious clone pMT-28 codifying the genomic RNA from FMDV C-S8c1 [48].

### 4.5. Virus Neutralization Test (VNT)

Serial two-fold dilutions of sera were incubated with 100 infection units—50% tissue culture infective doses (TCID50) of FMDV O/UKG 11/01, for 1 h at 37 °C. End-point titers were calculated as the reciprocal of the serum dilution that neutralized FMDV infection in 50% of the wells [28].

### 4.6. Detection of Specific Anti-FMDV Antibodies by ELISA

Total anti-FMDV antibodies were determined by ELISA. Briefly, 96-well plates (Nunc, Rochester, NY, USA) were coated with peptide B (1 µg) overnight at 4 °C. Duplicate three-fold dilution series of each serum sample were prepared in 50 µL, starting at 1/100. Pre-immune sera from peptide-immunized pigs and sera from non-immunized animals were used as negative controls. Specific antibodies were detected with HRP-conjugated protein A (Thermo Fisher, Rochester, NY, USA), diluted 1/4000. Color development was obtained after addition of 100 µL/well of TMB (Sigma Aldrich, St. Louis, MO, USA) and stopped by an equal volume of 1M H_2_SO_4_. Plates were read at 450 nm, titers expressed as the reciprocal of the last dilution giving the absorbance recorded in the control wells (serum at day 0) plus 2 SD.

### 4.7. PBMC Isolation and IFN-γ Detection by ELISPOT

Porcine peripheral blood mononuclear cells (PBMCs) were isolated by density gradient centrifugation using Histopaque-1077 (Sigma, St. Louis, MO, USA) and cryopreserved prior to assay. Vials were defrosted and resuspended in complete RPMI 1640 and incubated overnight at 37 °C, 5% CO_2_. Cell counting and viability were tested by Trypan blue staining. For the IFN-γ ELISPOT assay 2.5 × 10^5^ PBMCs were shed in triplicate wells of Immobilon-P plates (Merck Millipore, Burlington, MA, USA) coated with 5 µg/mL of anti-pig IFN-γ antibody (clone P2G10, Becton Dickinson, Franklin Lakes, NJ, USA). For in vitro antigen recall, PBMCs were stimulated with 25 μg/mL of the peptide used for pig immunization [49]. As positive control, PBMCs were incubated with 5 μg/mL of phytohaemagglutinin (Sigma) using cells incubated without antigen as negative control. After 48 h at 37 °C, 5% CO_2_, plates were washed and incubated with 2 µg/mL of biotinylated anti-mouse IFN-γ antibody (clone P2C11, BD) and HRP-streptavidin (BD). Antibody was visualized with 3-amino-9-ethyl carbazole (BD). The frequency of peptide-specific T cells in was expressed as the mean number of spot-forming cells/10^6^ PBMCs, with background values (number of spots in negative control wells) subtracted from the respective counts of stimulated cells.

### 4.8. Statistical Analyses

Differences among the peptide-immunized groups in FMDV-antibody titers and number of IFN-γ producing cells were analyzed by two-way ANOVA, followed by Tukey’s post-hoc comparisons tests. Values are cited in the text as means ± SD. All *p* values are two sided, and *p* values < 0.05 were considered significant. Statistical analyses were conducted using GraphPad Prism Software 5.0 (Graphpad Software, San Diego, CA, USA).

## Figures and Tables

**Table 1:** Synthetic peptides used in this study.

Peptide	FMDV Protein (Residues)	Sequence
B	VP1 (136–154)	PVTNVRGDLQVLAQKAART-amide
T	3A (21–35)	AAIEFFEGMVHDSIK-amide
B_2_T	VP1 (136–154), 3A (21–35)	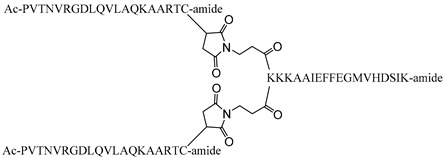

**Table 2:** Evidences for protection in immunized pigs.

Inoculum	Pig	VNT/IFNγ ^a^	Fever ^b^	Lesion Score ^c^	Protected ^d^	RNA ^e^
B_2_T (2 mg)	1	1.7/221	39.8 (6)	0	++	ND
	2	2.3/68	39.7 (6)	0	++	ND
	3	1.6/830	41.7 (7)	3 (7)	−	1.8 × 10^4^ (5)
	4	2.3/270	39.6 (7)	0	++	ND
	5	1.6/33	39.9 (10)	2 (10)	+	ND
B_2_T (0.5 mg)	6	1.3/696	No fever	0	++	ND
	7	1.5/10	No fever	0	++	ND
	8	2.3/332	No fever	0	++	ND
	9	2.6/1096	No fever	0	++	ND
	10	2/1093	39.6 (8)	5 (7)	−	10^8^ (3); 2 × 10^6^ (5)
Non-immunized	11		40.7 (5)	7 (5)	−	1.4 × 10^8^ (3); 4.5 × 10^6^ (5)
	12		No fever	7 (5)	− ^f^	1.1 × 10^8^ (3)

^a^ VNT and IFNγ spots/10^6^ PBMCs determined at day 21 and 15 post-immunization, respectively. ^b^ Temperature (°C) and (in parenthesis) day pi when maximum temperature registered. No fever: ≤39.0–39.5 °C. ^c^ Animals were monitored up to 10 days pc for lesions. Lesion score (maximum value of 7): 1 point/vesicle in foot (up to 4 points); 1 point/mouth, tongue or snout lesion; 1 point/>2 lesions of diameter ≥10 mm. In parenthesis, day pc when lesion(s) was first observed. ^d^ Animals were considered fully protected (++) if lesion score ≤ 1; partially protected (mild/delayed disease) (+) if lesion score ≤ 2, or non-protected (−) if lesion score > 2. ^e^ Detection of FMDV RNA in serum samples. ND: RNA not detected. The amount of RNA in positive animals are expressed as viral RNA copies (VRC)/mL serum. Detection limit: 5 × 10^3^ VRC/mL serum. The values from RNA positive animals are presented as VRC/mL serum. The day(s) post-challenge when RNA was detected is shown in brackets. ^f^ Animal died on day 5 pc.
